# Polycystic Ovary Syndrome and Eating Disorders—A Literature Review

**DOI:** 10.3390/jcm14010027

**Published:** 2024-12-25

**Authors:** Agata Góral, Klaudia Żywot, Wojciech Zalewski, Adam Jagodziński, Marek Murawski

**Affiliations:** 1 Faculty of Medicine, Wroclaw Medical University, 50-367 Wroclaw, Poland; klaudia.zywot@student.umw.edu.pl (K.Ż.); wojciech.zalewski@student.umw.edu.pl (W.Z.); 2 Clinical Department of Gynecologic Surgery and Oncology, Wroclaw Medical University, Borowska 213, 50-556 Wroclaw, Poland; a.jagodzinski@umw.edu.pl (A.J.); marek.murawski@umw.edu.pl (M.M.)

**Keywords:** polycystic ovary syndrome, eating disorders, hormonal imbalances, hyperandrogenism, insulin resistance, serotonin, leptin, ghrelin, cortisol, anxiety and depression

## Abstract

Polycystic ovary syndrome (PCOS) is a complex endocrine disorder that affects women of reproductive age and is characterized by hyperandrogenism, ovulatory dysfunction and polycystic ovarian morphology. PCOS is often associated with hormonal imbalances, metabolic dysfunction and comorbid psychiatric disorders, including eating disorders (EDs). The review identifies key hormonal factors—serotonin, leptin, insulin, ghrelin, kisspeptin and cortisol—and their roles in the pathophysiology of PCOS and associated psychiatric symptoms. Serotonin deficiency, commonly seen in PCOS patients, is associated with mood and eating disorders. Fluctuations in leptin, the satiety hormone, affect hypothalamic–pituitary–ovarian axis function and ovarian follicle maturation, increasing the risk of infertility. Elevated levels of kisspeptin in PCOS patients contribute not only to hormonal dysregulation but also to increased susceptibility to eating disorders such as bulimia and binge eating, likely due to its influence on the limbic system and glucose metabolism. Hyperinsulinemia and insulin resistance further impair reproductive and metabolic health, while promoting eating disorders such as binge eating and bulimia. Ghrelin and cortisol also emerge as significant factors. The review emphasizes the bidirectional relationship between PCOS and eating disorders, in which hormonal imbalances perpetuate psychiatric conditions, creating a vicious cycle. A multidisciplinary approach including gynecologists, endocrinologists, psychiatrists and nutritionists is recommended to ensure complex treatment. Early identification of those at risk through targeted screening and personalized interventions is key. Future research should focus on discovering the underlying hormonal mechanisms to improve treatment strategies and quality of life for women with PCOS.

## 1. Introduction

Polycystic ovary syndrome is a common disorder among women of reproductive age; it is, by definition, a normo-gonadotropic, normo-estrogenic state and is often associated with anovulation [1]. It is diagnosed by the Rotterdam criteria [2]. Those guidelines, established during an international congress held in Rotterdam in 2023, constitute that patients can be diagnosed with PCOS if there are at least two of the three following symptoms present: hyperandrogenism, oligo-/anovulation and typical sonographic ovary image.

The diagnostic criteria for PCOS, particularly during adolescence, are controversial because many features used in diagnosing adult women, such as acne, irregular menses and polycystic ovary morphology, can be normal physiological characteristics of puberty [3].

While approximately 30 genes contribute to the development of PCOS, various other factors play important roles in the development of this complex disease [4,5]. Numerous hypotheses concerning the etiopathogenesis of PCOS tend to complement one another. The most widely recognized theory is that the development of PCOS is due to insulin resistance and hyperinsulinemia, which subsequently lead to hyperandrogenism [6]. Polycystic ovary syndrome can be associated with metabolic dysfunction, obesity, psychiatric conditions, infertility, sleep disorders, sexual dysfunction and long-term cardiovascular disease [7,8,9,10,11].

Compared to women without PCOS, women with PCOS are more likely to have eating disorders. The most commonly reported are bulimia nervosa and binge eating. However, anorexia nervosa is rarely diagnosed [12]. Binge eating is characterized by episodes of out-of-control consumption of a large amount of food in short period of time [13]. Bulimia nervosa, on the other hand, is a condition in which an episode of binge eating is followed by compensatory behaviors to prevent weight gain, such as self-induced vomiting, use of laxatives or diuretics, exhausting physical activity or fasting [14]. The prevalence of eating disorders in this group of patients is up to 62%. Bulimia nervosa can be observed in 12%, and binge eating may occur in 42% of women [15]. Women with PCOS are also more likely to have sleep disorders such as hypersomnia and obstructive sleep apnea [7].

In this study, polycystic ovary syndrome is going to be looked at, with a focus on imbalances in hormones and neurotransmitters, showcasing their importance in the development of eating disorders. The main neurotransmitter that will be expanded upon is serotonin, as it was discovered that its levels vary between healthy women and women with PCOS. Of many hormones that play an important role in the mechanism of PCOS and associated eating disorders, we choose to point out leptin and its pathological fluctuations, leading to the disturbance of follicle maturation; insulin, as hyperinsulinemia correlates with PCOS; ghrelin (pointing out the correlation between its influence of hunger leading to the development of obesity, which is proven to be one of the major factors of developing PCOS and associated eating disorders); and cortisol, specifically its imbalance in the context of follicular development and stress as a psychological mechanism, which is important to understand the correlation between psychiatric disorders including eating disorders and PCOS.

## 2. Methodology

We performed a literature review on eating disorders in women with PCOS through searches in the PubMed, Web of Science and Scopus databases. The phrases used to search for articles included “polycystic ovary syndrome” or “PCOS”, “eating disorders”, “eating disorders in PCOS” “psychiatric disorders in PCOS”, “hormonal changes”, “binge eating” and “bulimia nervosa”. The articles analyzed were published between 1993 and 2024, focusing on studies no older than five years. Articles in languages other than English, case reports and works without open access were excluded. No restrictions were imposed on the countries where the studies were conducted. Duplicate search results were removed. Additionally, we conducted a manual review of the reference lists from the different studies included in this review to identify any supplementary publications that might be able to contribute valuable insights to the current paper.

## 3. Diagnostic Criteria

PCOS was first described in 1935 by Stein and Leventhal as an endocrinological–metabolic disorder with an unknown etiology. Nowadays, the origins of PCOS are much more recognized, and through the years, diagnostic criteria have been defined.

The first criteria, called “classic”, were completed by the NIH (National Institute of Child Health and Human Development) in 1990. At that time, the PCOS criteria consisted of the presence of clinical and/or biochemical signs of hyperandrogenism and oligomenorrhea or amenorrhea. In contrast to present diagnostics, ultrasonography did not play such an important role. Ultrasonographic studies to search for evidence of PCOS ovaries were considered as a suggestion, but they were not a necessary point of diagnosis.

The discussion was continued in 2003 in Rotterdam, which gave the name to the well-known “Rotterdam criteria”, extended with ultrasonography but deprived of hyperandrogenism [16]. According to the criteria, to identify PCOS by ultrasonography, it is necessary to observe 12 or more follicles with a diameter between 2 and 9 mm and/or an enlarged volume of the ovary above 10 cm3 in one or both ovaries. This examination should be performed between the third and fifth days of the menstrual cycle, while in amenorrheic women or patients with infrequent menstruation, the date of examination does not matter. However, the primary Rotterdam criteria were not highly specific: with the increase in availability of transvaginal ultrasonography, it was found that nearly 20–30% of regularly cycling women fulfilled the criteria [17].

Until 2006, women were allowed to be diagnosed with PCOS without hyperandrogenism, which had been previously the main requirement to recognize PCOS. In 2006, the AES (Androgen Excess Society) again made hyperandrogenism a requirement, establishing the presence of hirsutism and/or biochemical hyperandrogenism as a necessary PCOS criterion. Due to the existence of three different classifications, the NIH started to work on standardizing PCOS criteria in 2012. Cooperation between PCOS specialists resulted in the extension of the Rotterdam criteria to include the condition of hyperandrogenism. Broader Rotterdam criteria continue to be used by gynecologists today. Nowadays, a patient is qualified with PCOS if two of the following three conditions are fulfilled: (1) clinical or biochemical hyperandrogenism, (2) oligo-/anovulation and (3) polycystic-appearing ovarian morphology (PCOM). Biochemical hyperandrogenism is elevated total or free testosterone. The free androgen index (FAI), which is calculated by taking total testosterone divided by the sex hormone binding globulin (SHBD) level and then multiplying by 100, is considered a reliable marker of estimated free testosterone. Bioavailable testosterone (free testosterone plus albumin-bound testosterone) may also be used to diagnose hyperandrogenism [16]. The modified Ferriman–Gallwey scale, which is the gold standard for diagnosing hirsutism, is used to evaluate clinical hyperandrogenism. Hair growth is evaluated in nine androgen- sensitive regions of the body and, depending on the intensity, is graded on a scale of 0 to 4 points, where 0 points means no hair and 4 points means excessive hair growth. A score above 8 is considered as hirsutism [18]. Among the patients with PCOS, 75–90% present hyperandrogenemia, and approximately 60–76% of women experience hirsutism [19]. By definition, oligo-/anovulation is the situation in which a patient has cycles more than 35 days apart or has fewer than eight cycles per year. Polycystic ovarian morphology is defined as ≥20 follicles per ovary and/or an ovarian volume of ≥10 cm^3^ on one or two ovaries, using transvaginal ultrasound, when other medical causes are excluded [16].

### 3.1. Criteria for Adolescents

There is no general consensus on how PCOS should be defined in adolescents because of the shared common features between pubertal development and PCOS. For example, multifollicular ovaries can be found in approximately 26% of adolescents [20]. Moreover, during puberty, relative ovarian volume is greater than that of adults. Limited evidence suggests that two years after menarche, the threshold for ovarian size is similar to that of adults. Despite the fact that menstrual dysfunction is a common feature of normal reproductive maturation [21], prolonged adolescent oligomenorrhea at the age of 14–19 is predictive of persistent ovarian dysfunction in following years [22]. Therefore, diagnostics of adolescent girls should be performed differently from that of mature women.

The diagnosis should be deferred at least for two years after menarche. In addition, it should be primarily based on clinical and biochemical signs of hyperandrogenism and irregular periods [20].

Hyperandrogenism should be one of the main criteria for diagnosing adolescent women, because total and free testosterone levels in adolescents 1 to 2 years after menarche are comparable to those in adults [23,24]. By the age of 18, the modified Ferriman–Gallwey hirsutism scores are likely to be the same as in an adult. In a study of 633 unselected women presenting for a pre-employment physical examination, mostly aged 18 through 45 years, the modified Ferriman–Gallwey score was established to be unassociated with age [25]. Therefore, it is likely that adult criteria for hyperandrogenism and ovarian volume, but not follicular count, could be used in adolescents who are two years past menarche [26]. Two sets of adolescent PCOS criteria have been suggested.

One was proposed by an ESHRE/ASRM working group and goes as follows: the patient should display clinical or biochemical hyperandrogenism (increased serum androgens and/or progressive hirsutism), oligo-/anovulation (oligo-/amenorrhea for at least two years, or primary amenorrhea by the age of 16) and polycystic ovarian morphology (ovarian volume >10 cm^3^). All three criteria must be met, with exclusion of other etiologies [27].

The other set was established by a clinical practice guidelines committee of the Endocrine Society [28] and goes as follows: the patient should display clinical or biochemical hyperandrogenism (increased serum androgens and/or progressive hirsutism), persistent oligo-/anovulation (oligo-/amenorrhea for at least two years, or primary amenorrhea by age of 16 years) and polycystic ovarian morphology (ovarian volume >10 cm^3^). Two of the three criteria are required, with exclusion of other etiologies.

Girls who do not fulfill all the diagnostic criteria should be treated symptomatically. It was established that the majority of adolescent girls who present early signs of the syndrome will develop PCOS clearly by the age of 18 [29].

### 3.2. Phenotypes of PCOS

Despite the criteria that are present in PCOS patients and are common to the disease, some patients will present with only some of the symptoms. This has made it possible to distinguish phenotypes and subtypes of this disease.

According to the Rotterdam criteria, three main features of women with PCOS are hyperandrogenism (HA), ovulatory dysfunction (OV) and/or polycystic ovary morphology (PCOM). These three symptoms make it possible to classify four different phenotypes, labeled A–D. Phenotype A contains all the features included in the Rotterdam criteria. In phenotype B, there are symptoms of hyperandrogenism and ovulatory dysfunction. Phenotype C is manifested by hyperandrogenism and polycystic ovary morphology. Phenotype D consists of ovulatory dysfunction and polycystic ovary morphology [30].

Phenotypes A and B are often called “classic” PCOS. Women with these two types experience more irregular menstrual cycles compared to types C and D. They are also more likely to display obesity, insulin resistance, dyslipidemia and fatty liver disease compared to women with other PCOS types [31,32].

Phenotypes C and D were introduced by the 2003 Rotterdam criteria. Phenotype C is called “ovulatory” PCOS. Patients with this type of the disease have an intermediate risk of metabolic syndrome and intermediate androgen levels compared to the “classic” type and phenotype D PCOS [33]. Phenotype D is called “nonhyperandrogenic” PCOS. Patients with this phenotype have normal androgen levels and the lowest risk of metabolic disorders compared to classic PCOS [31]. However, in the Turkish study, women with phenotype D had low-density lipoprotein cholesterol levels that were higher compared to patients with PCOS phenotype C [34].

## 4. Serotonin

Serotonin (5-HT) is a neurotransmitter that affects behavior, mood, memory and many other activities of the brain. Serotonin is mainly associated with psychiatric and neurological disorders such as depression, anxiety or eating disorders. An amino acid—tryptophan—is required for the synthesis of serotonin. It undergoes hydroxylation by tryptophan hydroxylase and decarboxylation by 5-hydroxytryptophan decarboxylase [35].

In women with PCOS, one of the elevated parameters is luteinizing hormone (LH), which is regulated by gonadotropin-releasing hormone (GnRH). One of the many factors that can regulate the pulsatile release of GnRH/LH in the body is serotonin, which can inhibit the pituitary secretion of LH [36].

Serum levels of 5-HT and 5-hydroxyindoleacetic acid (5-HIAA) have been shown to be lower in women with PCOS compared to a group without PCOS [37]. A study involving rats also showed that serotonin levels were significantly reduced in the brain tissue of animals with PCOS. Similarly, expression of the 5-HT1A receptor was found to be reduced in the same group of animals [36]. The above data support the theory that women with PCOS have a higher risk of developing psychiatric disorders due to reduced serotonin levels. Studies have also shown that patients with anorexia nervosa have lower levels of 5-HIAA in cerebrospinal fluid compared to healthy controls, which may be related to selective food intake, leading to a deficiency of tryptophan, which is needed for serotonin synthesis [38]. In addition, long-term food restriction reduces the activity of serotonin transporters [39].

Dysregulated levels of neurotransmitters are responsible for the coexistence of psychiatric disorders in this group of patients. A study using the Hospital Anxiety and Depression Scale revealed that women with PCOS have an increased risk of clinically significant anxiety and depression than women without PCOS. Anxiety disorders were observed in 41% of PCOS patients participating in the study, while depressive symptoms were observed in 12% of the same participants [40].

Along with the hormonal changes, PCOS can also cause appearance changes such as hirsutism, androgenetic alopecia, acne, seborrhea or weight gain [41]. According to beauty standards, these symptoms are highly undesirable among women. Patients suffering from PCOS manifest lower self-esteem and concern about their weight [8,42]. Both hirsutism and high BMI can lead to body dissatisfaction and eating disorders [43,44,45]. The prevalence of eating disorders in women with hirsutism was found to be 36.3% [46].

## 5. Leptin

Leptin is a hormone of satiety produced by adipocytes. Its concentration is proportional to the content of triacylglycerols in the white fat tissue. Leptin affects LEPR receptors in the hypothalamus, crossing the blood–brain barrier, but also affects NLPR3 receptors in the ovary, associated with ovulation, thus inhibiting follicle development and leading to infertility.

Stimulation of the hypothalamus not only plays a role in reducing food intake and increasing thermogenesis but also stimulates the effect on the hypothalamic–pituitary–ovarian axis, which leads to the secretion of the hormones GnRH and LH. These two hormones have a significant role in the maturation of oocytes. Studies on mice have shown that individuals with physiological concentrations of leptin reached the highest levels of both hormones [47]. Either mice with increased levels of leptin that were overfed or mice with decreased levels of leptin after starvation had reduced LH and GnRH secretion.

Research on the effect of leptin on ovarian receptors can be found as well [48]. Studies on mice have shown that high levels of leptin, acting through NLPR3 receptors, inhibit the transition from primary to secondary follicles, but lower levels of leptin stimulate this process. Bulimia nervosa and binge eating disorder lead to overeating and consequently reduce amounts of triacylglycerols in the fat tissue, stimulating leptin, which lowers LH and GnRH levels and inhibits the maturation of follicles with ovarian leptin receptors. However, anorexia nervosa, rapid weight loss and starvation also cause reduced levels of LH and GnRH and inhibition at the primary follicle stage in the case of very low leptin levels.

Leptin imbalances occur during the development of obesity and PCOS, showcasing the clear correlation. An interesting study published in 2020 dove into the mechanism of this pathology. It was established that leptin levels significantly increased compared with those of controls. This was associated with an upregulated Th1 cell proportion and IFN-γ level. In vitro, the Th1 cell proportion increased after leptin treatment of PBMCs from PCOS patients. Furthermore, for KGN cells, the percentage of live cells decreased and the percentage of apoptotic cells later increased after IFN-γ treatment. This shows that leptin takes part in PCOS development by inducing the expression of IFN-γ and points out the significance of the connection between leptin and inflammation in PCOS [49]. A particularly interesting fact is that increased levels of leptin as well as leptin resistance in PCOS occur not only in obese patients but also in slim ones [50].

## 6. Insulin

Insulin resistance (IR) is a condition in which cells are insensible to insulin. The main impact on the origins of IR is hyperinsulinemia. An elevated level of insulin is often connected with the image of a patient with a diet poor in nutrients, especially filled with products with high glycemic index, and engaging in negligible physical activity. Therefore, high levels of insulin will be seen often in women who suffer from bulimia nervosa as a consequence of binge eating, characterized as eating huge quantities of usually unhealthy, processed food. Despite the compensation mechanisms, such as vomiting, a high concentration of insulin in the bloodstream is preserved.

Insulin, when activating the insulin receptor, starts a biochemical reaction pathway via mTORC1 kinase and S6K kinase, which leads to the internalization of the insulin receptor from the cell membrane. In conclusion, despite the high concentration, insulin cannot cause any effects [51]. In 2009, a research was carried out examining the correlation between short-term overeating and insulin resistance. The study involved 18 young people who were subjected to binge eating for four weeks. After the research period, an adipose tissue biopsy was taken from each individual and given analysis and study in vitro. The quantity of insulin receptors was found to be reduced by about 40% [52].

The correlation between insulin resistance and PCOS was discovered by Burghen in 1980. It is influenced by several consequences caused by high insulin concentration in the bloodstream. Insulin receptors are numerous in the hypothalamic–pituitary–ovarian axis, and their binding to insulin leads to many factors causing PCOS. First of all, insulin activates the hypersecretion of androgens from ovaries and adrenal glands. As a consequence, the testosterone concentration in the bloodstream is increased. Hyperinsulinemia also stimulates ovarian theca cells to produce more LH, which, in the pathway, leads to the release of GnRH, leading to excess androgens [53]. Insulin can also directly affect the maturation of oocytes. Insulin in physiological concentrations has a stimulatory effect on the maturing oocytes, whereas hyperinsulinemia has a negative impact on the developmental competence of them.

It is estimated that 70% of PCOS patients suffer from insulin resistance, but that figure is affected by frequent referral bias. It might be a common misconception to think of insulin resistance in those women as an effect of obesity, which is a prevalent trait among them, but it is only partially the truth. There is research supporting the claim that insulin resistance in PCOS patients in intrinsic [54,55]. The impact of BMI on the IR of the women with PCOS is greater than in women not suffering from PCOS. Patients with normal BMI and PCOS also display insulin resistance more frequently than overweight women without PCOS [55]. A study from 2005 focused on the exact pathogenesis of that phenomenon. It was established that increased phosphorylation of IRS-1 Ser^312^ may contribute to a signaling defect [56]. One issue is the role that hyperinsulinemia plays in the androgen overproduction which is one of the core factors in developing PCOS and the fact that normalizing BMI will not always lead to eradication of IR and following expected lowering levels of androgens [57].

Apart from the influence on the reproductive system insulin also has many systemic effects, many of which are especially significant to women suffering from eating disorders. Intense hunger and craving for sugar may impede refraining from bingeing, leading to more frequent binge eating. Not only consumption of large numbers of calories caused by bulimia nervosa but also hyperinsulinemia on its own is causing weight gain via insulin resistance; glucose cannot be metabolized by cells such as adipocytes or myocytes, and it is transformed into triglycerides and stored in fat tissue.

Other hyperinsulinemia symptoms, such as difficulty in concentration, feeling anxious and lack of motivation lead to deterioration of mental well-being and depression, which is a significant element of both bulimia nervosa and anorexia nervosa. As can be seen, hyperinsulinemia can facilitate the development of both bulimia nervosa and binge eating. In the course of these diseases, it may lead to hyperinsulinemia, which causes insulin resistance, an important clinical component of PCOS.

## 7. Kisspeptin

Kisspeptin is an important hormone for both reproductive system and glucose homeostasis produced by arcuate nucleus and anteroventral periventricular nucleus of the hypothalamus [58]. The kisspeptin receptor KISS1R is present on over 90% of gonadotropin-releasing hormone neurons and stimulates pulsate GnRH release [59]. Studies showed that the concentration peaks of kisspeptin and GnRH overlap each other, which further proves that Kisspeptin affects GnRH secretion. GnRH effects release of luteinizing hormone (LH) and follicle-stimulating hormone (FSH), which play primary roles in fertility and the menstrual cycle.

Even though most KISS1Rs are located in hypothalamus, they have also been found in the limbic system, specifically the amygdala, thalamus, hippocampus and cingulate cortex [60]. The limbic system is an anatomical area of brain which is responsible for emotion, memory, ambitions and behavior. Studies have shown that kisspeptin has an anxiogenic, but antidepressive effect on rats. Localization of those receptors in the limbic system may be interesting in context of this publications. Both bulimia nervosa and anorexia nervosa’s neural roots are related to a dysfunctional limbic system. Some studies have shown that kisspeptin serum levels are increased in women with PCOS. This increase brings two important consequences: constant stimulation for secretion of GnRH leads to constant secretion of LH and FSH, which causes transformation of physiological ovaries into polycystic ovaries and excessive androgen secretion, and it consequently fuels deepening of symptoms and worsens the prognosis [61]. Some studies show that kisspeptin may decrease food intake via stimulation of POMC/CART and inhibition of NPY/AgRP; therefore it could be called an anorexigenic factor, which is a bad prognostic factor in eating disorders [62].

Kisspeptin receptors have also been found in the pancreas, with most of them in the endocrine part of the pancreas and very few in the exocrine pancreas [63]. Both in vitro and in vivo studies have shown that kisspeptin at high levels has a stimulatory effect on glucose-stimulated insulin secretion [64,65]. However, kisspeptin cannot increase insulin secretion on its own; there must be an adequate concentration of glucose. Combining the facts that women with bulimia nervosa or binge eating have high glucose levels due to compulsive eating, if they additionally have PCOS and high serum kisspeptin levels, a synergistic effect is created in them, leading to insulin resistance, which is part of the pathogenesis of PCOS.

## 8. Ghrelin

Ghrelin is a peptide hormone produced by the enteroendocrine cells A of the stomach during starvation. Its functions include the regulation of appetite and secretion of growth hormone. In addition, ghrelin is responsible for regulating gastric acid secretion, gut motility, endocrine and exocrine pancreatic secretion and ovarian function [66,67]. There have been studies showing that women with PCOS have lower levels of ghrelin [68,69,70]. Similarly, ghrelin levels decrease in people with obesity and binge eating, while an increase in ghrelin levels has been observed in people with anorexia nervosa [71,72,73]. There are also studies that report no changes in ghrelin levels in PCOS patients compared to non-PCOS patients [74]. However, there is a study showing that obese women with PCOS compared to obese women without PCOS, non-obese women with PCOS and without PCOS had significantly lower levels of ghrelin. The same study shows that non-obese PCOS patients had similar ghrelin levels to non-obese patients without PCOS [75]. This can suggest that decreased ghrelin level in a group of PCOS patients is mostly connected with being overweight than with the polycystic ovary syndrome itself. Although the direct effect of ghrelin on controlling the function of the reproductive system has rarely been evaluated, the hormone can cause increased appetite and overeating, leading to increased body fat, which is a risk factor for PCOS [68].

## 9. Cortisol

Cortisol is a steroid hormone from the group of glucocorticoids and is also called a stress hormone. It is produced by the zona fasciculata of the cortex of the adrenal gland, as well as by other tissues in lower amounts. Many women affected by PCOS have increased concentrations of cortisol in the bloodstream [76]. This hormone has a significant impact not only on glucose and lipid metabolism and the immune response but also on the functioning of the reproductive system. Its suppressing effect has been shown either in the hypothalamus-pituitary-ovary axis or the ovary itself. High concentrations of CRH (corticotropin-releasing hormone) inhibit the secretion of GnRH and increase the production of prolactin, both of these factors decrease the ability of ovaries to produce the appropriate level of hormones necessary for oogenesis [1]. Studies on mice have shown that oogenesis was impaired in individuals subjected to chronic stress and consequently elevated cortisol levels. Researchers observed the inhibition of both secondary and antral ovarian follicle development and increased ovarian follicle atresia, which may be due to the inhibited expression of growth and differentiation factor 9 (GDF 9). Additional studies also showed that the expression of brain-derived neurotrophic factor (BDNF) in antral follicles is reduced. The number of atretic antral follicles increased. Furthermore, the development of follicles was impaired due to the absence of corpus luteum and pyknotic granuloma cells. Moreover, cortisol triggers an oxidative stress, which also reduces reproductive potential.

Stress is an important factor in the development of depressive and eating disorders. Lower serotonin levels may also be related to continued exposure to cortisol, whose levels are elevated in a group of women with PCOS [77]. Stress is known to be a major provoking factor in binge eating, which is the most commonly described eating disorder among women with PCOS [78]. This eating pattern may be a coping mechanism to deal with the psychological stressors associated with PCOS. Stress is also an element that negatively affects satisfaction with body image and self-esteem [79].

## 10. Summary

It is important to remember that women suffering from PCOS are especially prone to the development of eating disorders, due to imbalances in levels of their hormones and neurotransmitters. Those imbalances can lead to the development of eating disorders, which furthermore contribute to the development of hormonal imbalances, that presents itself as a vicious cycles. Moreover, the phenotype of patients with PCOS causes degradation of their quality of life and perception of their body image, which leads to an increase in the occurrence of eating disorders.

In addition to its well-known effects on mood, serotonin is one of the factors that inhibit the pulsatile release of LH. Eating disorders, specifically anorexia nervosa, may also contribute to lower serotonin levels, due to a deficiency of its precursor. Patients without eating disorders but diagnosed with PCOS also show lower levels of serotonin, making them more prone to psychiatric disturbances such as depression, anxiety or eating disorders. It is important to pay attention to the general well-being of patients, including early symptoms of depressive disorders.

Leptin plays an important role in the mechanism of PCOS through NLPR3 receptors in the ovary and stimulation of the hypothalamic—pituitary—gonadal axis. The excess of leptin occurring during the eating disorders displaying overeating behaviors causes infertility, due to inhibiting follicle development. However, the decreased levels of leptin during fasting, which can be observed in eating disorders such as anorexia, also lead to infertility, due to reduced levels of LH and GnRH and inhibition at the stage of the primary follicle. It is important for patients with PCOS to maintain a well-balanced and properly calorie-rich diet to avoid imbalances of leptin.

Insulin resistance also plays a significant role in the development of polycystic ovary syndrome, where high insulin levels lead to excessive androgen production. In addition, hyperinsulinemia impairs oocyte maturation and has a negative impact on mental health, which can exacerbate bulimia nervosa and contribute to the further development of insulin resistance and PCOS. Patients with insulin resistance should be subjected to a diet, that takes into consideration the low glycemic index, regular physical activity and check-ups to control carbohydrate metabolism.

Women with PCOS often have elevated cortisol levels, which affects not only glucose and lipid metabolism but also the functioning of the reproductive system. Cortisol inhibits the hypothalamic–pituitary–ovarian axis and oogenesis, which can lead to impaired ovarian follicle development and reduced reproductive potential. In addition, stress and associated elevated cortisol levels can lead to eating disorders such as binge eating, which is the most common eating disorder in women with PCOS.

When it comes to ghrelin, studies are not entirely consistent, but most suggest that lower ghrelin levels in PCOS patients are related to obesity comorbidities, rather than polycystic ovary syndrome itself. People with eating disorders also show fluctuations in ghrelin levels. Eating regularly can help to stabilize its levels.

Elevated kisspeptin levels, common in PCOS, contribute to hormonal imbalances, ovarian dysfunction and excess androgen production. Kisspeptin also interacts with the brain’s limbic system, linking it to emotional regulation and behaviors associated with eating disorders such as bulimia and binge eating. Furthermore, its role in glucose metabolism and insulin secretion may exacerbate insulin resistance in PCOS patients, especially when combined with disordered eating behaviors.

The current stage of research leads us all to agree that eating disorders are doubtless more common among women with PCOS and must be taken into consideration during the treatment of such patients. The ideal solution would be to select a group of patients suffering from PCOS who are at risk of developing eating disorders and observe them. For this purpose, screening methods in the form of questionnaires can be used. Survey questions should be focused on signs of depression according to the Rosenberg scale, presenting the possibility of evaluating self-esteem and medical interviews, including previous history of eating as well as psychiatric disorders. It is also important to pay attention to the patient’s lifestyle, including stimulants such as alcohol and smoking. In case of such a complex condition as PCOS, it is crucial to introduce interdisciplinary cooperation between gynecologists, endocrinologists, psychiatrists, psychologists and dietitians to ensure the optimal care of patients with both PCOS and eating disorders. If patients are already showing signs of eating disorders, it should be a priority to diagnose them as early as possible to prevent the development of further hormonal imbalances. All the research focused on the exact hormonal grounds of eating disorders should be continued due to many unanswered questions regarding this issue.
